# Effects of intranasal long-acting insulin pretreatment on postoperative delirium and the NLRP3/caspase-1/IL-1β pathway in older patients with esophageal cancer

**DOI:** 10.1186/s41232-026-00418-4

**Published:** 2026-04-18

**Authors:** Yong Zhang, Wei Wu, Zuling Zhong, Yinghai Liu, Gu Gong, Qingqing Huang

**Affiliations:** Department of Anesthesiology, The General Hospital of Western Theater Command, 270 Tianhui Road, Rongdu Avenue, Jinniu District, Chengdu, Sichuan 610083 China

**Keywords:** Intranasal administration, Long-acting insulin, Postoperative delirium, NLRP3 inflammasome, Elderly patients, Esophageal cancer

## Abstract

**Background:**

Insulin exhibits neuroprotective and anti-inflammatory properties. Preoperative intranasal insulin preconditioning is a potential strategy to prevent postoperative delirium (POD), but prior studies mainly used rapid-acting formulations. This investigation focused on intranasal long-acting insulin, which ensures sustained central nervous system exposure, in elderly patients undergoing radical esophagectomy. We assessed its impact on POD incidence and the NLRP3/caspase-1/IL-1β pathway.

**Methods:**

Sixty older patients scheduled for elective radical esophagectomy were randomized into two groups. The intervention group (*n* = 30) received a single intranasal dose of long-acting insulin (30 U) 1 day preoperatively, while the control group (*n* = 30) received an equivalent volume of physiological saline. POD was evaluated using the Confusion Assessment Method for the ICU on postoperative days 1, 2, and 3. Peripheral blood samples were collected before surgery and postoperatively to measure IL-1β concentrations and NLRP3/caspase-1 mRNA expression in mononuclear cells.

**Results:**

Compared to controls, long-acting insulin pretreatment significantly reduced POD incidence (16.7% vs. 46.7%, *P* = 0.012) and suppressed the postoperative rise in peripheral IL-1β levels (*P* < 0.05). In addition, NLRP3 and caspase-1 mRNA expression were notably lower in the insulin group during the postoperative period (*P* < 0.05). Correlation analysis revealed that in the control group, increases in NLRP3 mRNA, caspase-1 mRNA, and IL-1β levels on postoperative day 1 were significantly associated with the development of POD (*P* < 0.05). In contrast, no such significant correlations were found in the insulin intervention group.

**Conclusion:**

Preoperative intranasal long-acting insulin effectively decreases POD incidence in the first 3 days after radical esophagectomy in older patients. This protective effect may be associated with the observed sustained downregulation of the peripheral NLRP3/caspase-1/IL-1β signaling pathway, which consequently weakens the link between early postoperative inflammation and delirium. This emphasizes the advantage of long-acting formulations for continuous neuroprotection during the critical postoperative phase.

## Introduction

Esophageal cancer (EC) is a common gastrointestinal malignancy in older individuals, with radical surgery remaining the primary treatment approach [1]. However, older patients are more prone to postoperative complications, particularly postoperative delirium (POD) due to age-related physiological reserve decline [2]. Postoperative delirium is formally defined as an acute, fluctuating disturbance in attention, awareness, and cognition that develops shortly after a surgical procedure [3]. Its occurrence in older surgical patients is multifactorial, involving a complex interplay of predisposing factors (e.g., advanced age, pre-existing cognitive impairment) and precipitating factors (e.g., surgical trauma, anesthesia, and pain) [4]. As a result of these factors, POD can delay postoperative recovery, prolong hospital stay, increases healthcare costs, and is associated with a significantly increased risk of adverse events such as falls, tube dislodgement, and infection. In severe cases, POD may lead to long-term cognitive impairment [5].

Recent evidence has shown that insulin exerts significant neuroprotective and anti-inflammatory effects beyond its role in glycemic control. These include enhancing synaptic plasticity, inhibiting neuronal apoptosis, and suppressing pro-inflammatory cytokine release [6, 7]. Intranasal administration of insulin, a non-invasive route with rapid access to the central nervous system, has shown considerable advantages in both animal and clinical studies. This method bypasses the blood-brain barrier and delivers insulin directly to the brain tissue via the olfactory and trigeminal perivascular pathways, effectively targeting key regions such as the hippocampus [8]. Compared with intravenous administration, intranasal delivery avoids peripheral accumulation and reduces the risk of systemic hypoglycemia. It also enables more efficient and direct targeting of the CNS, enhancing its neuroprotective potential [9]. Therefore, intranasal insulin pretreatment is emerging as a promising strategy to reduce POD risk in older patients undergoing esophagectomy [10]. We previously demonstrated that multiple preoperative intranasal rapid-acting insulin administrations (30 U twice daily for 2 days preoperatively) significantly reduced the incidence of POD and peripheral levels of interleukin-1β (IL-1β) in older patients with gastrointestinal tumors [11]. However, the requirement for repeated dosing in such regimens may pose challenges for clinical implementation. In contrast, the use of long-acting insulin formulations, such as insulin detemir, offers the potential for sustained central nervous system exposure with a simplified single-dose preoperative administration, which may improve practicality and adherence while maintaining efficacy. The distinct pharmacokinetic profile of long-acting insulin could lead to more prolonged modulation of neuroinflammatory pathways, a hypothesis that remains to be rigorously tested.

The NLRP3/caspase-1/IL-1β signaling pathway is a key mediator of inflammatory responses and plays an important role in POD pathogenesis [12]. Perioperative stressors, such as infection, surgical trauma, and tissue injury, can activate the NLRP3 inflammasome, which in turn promotes caspase-1-mediated cleavage of pro-IL-1β and pro-IL-18 into their active forms. These cytokines may then cross a disrupted BBB, activate microglia and astrocytes, and initiate a neuroinflammatory cascade. The cascade contributes to synaptic dysfunction, altered glutamate metabolism, and neuronal injury—ultimately impairing cognitive functions such as learning, memory, and attention [13, 14].

Therefore, elucidating how intranasal insulin modulates the NLRP3/caspase-1/IL-1β axis may provide crucial mechanistic insights into its role in preventing POD and support the development of individualized perioperative immunomodulatory strategies. Building upon our previous clinical findings [10, 11], the present study was conducted in an independently recruited cohort of elderly esophageal cancer patients, using a single 40 U preoperative dose of intranasal long-acting insulin detemir [15]. This regimen contrasts with the multi-dose, rapid-acting insulin approach used in prior studies and aims to evaluate the feasibility and efficacy of a simplified dosing strategy. Furthermore, unlike our earlier investigations that primarily focused on serum inflammatory or neurodegenerative biomarkers, this work further examined the dynamic expression of NLRP3 and caspase-1 mRNA in peripheral blood mononuclear cells (PBMCs), aiming to elucidate the molecular mechanism by which intranasal long-acting insulin downregulates the NLRP3/caspase-1/IL-1β inflammasome pathway and exerts neuroprotective effects.

## Materials and methods

### Ethics statement

This study adhered to the principles of the Declaration of Helsinki, followed the Comprehensive Standards for Trial Reporting (CONSORT) guidelines, and was approved by the Ethics Committee of the General Hospital of Western Theater (Ethics Approval No. 2019ky64). The study was registered in the Chinese Clinical Trials Registry (http://www.trialregister.nl, date: 22/02/2022; Number: ChiCTR2200056906). All participants provided written informed consent.

### Participants

We enrolled sixty older patients who underwent radical resection of esophageal cancer at our hospital from February 2022 to October 2022. The inclusion criteria were as follows: age ≥ 65 years, American Society of Anesthesiologists (ASA) grade I–III, body mass index (BMI) ≤ 28 kg/m^2^, planned radical esophagectomy under general anesthesia, and provision of written informed consent. The following exclusion criteria applied: current participation or participation in another interventional clinical trial within the past 3 months, patients with contraindications to intranasal insulin administration (such as nasal defects and lesions), history of hypertension or diabetes, history of insulin allergy, history of alcoholism or drug abuse, inability to communicate before surgery (coma, deep dementia, language impairment, or severe visual and hearing impairment), low Mini-Mental State Examination (MMSE) score (defined as <17 for illiterate individuals, <20 for those with a primary school education, and <24 for those with a secondary school [including technical secondary school] education), severe liver and kidney dysfunction, and cardiovascular and cerebrovascular diseases. The exit criteria were as follows: insulin administered non-nasally during the study, reoperation or endotracheal intubation performed within 3 days post-surgery, unexpected adverse events (including drug allergies, anesthesia, and surgical complications), and loss to follow-up.

### Randomization, interventions, and blinding

Computer-generated randomization tables were employed for patient allocation. The resulting assignment sequence was concealed using sequentially numbered, sealed, opaque envelopes. Participants were randomly assigned to one of two groups: the control group (Group C; *n* = 30) received 0.3 ml of 0.9% saline solution, and the intervention group (Group I; *n* = 30) received 0.4 ml of long-acting insulin detemir (30 U), both administered via the nasal route. At 17:00 on the day preceding surgery, all patients received their respective preparations through a nasal mucosal nebulization device with a syringe (Wuxi Meihao Life Technology Co., Ltd., Jiangsu, China).

The study medications were prepared by an independent researcher not involved in patient anesthesia or outcome assessment. Long-acting insulin (3 ml, 300 U) was supplied by Novo Nordisk (China) Pharmaceutical Co., Ltd (Tianjin, China). To ensure blinding, participants, anesthesiologists, surgeons, nursing staff, follow-up personnel, and data analysts remained unaware of group assignments throughout the study period. The allocation details were disclosed only after database lock and completion of all statistical analyses.

### Method of anesthesia

Before anesthesia, we established peripheral venous access, performed electrocardiography, and attached a pulse oximetry probe to the patient. Blood pressure was monitored via radial artery puncture and catheterization. We performed central venous puncture and catheterization to monitor central venous pressure and guide transfusion. Bispectral index (BIS) and regional oxygen saturation were measured intraoperatively.

Anesthesia induction: sufentanil 0.4 μg/kg, etomidate 0.3 mg/kg, and rocuronium 0.6 mg/kg were administered as muscle relaxants. Mechanical ventilation was performed via endotracheal intubation under guidance by a video laryngoscope. The tidal volume was 8–10 ml/kg (6–10 ml/kg for pneumothorax), with a positive end-expiratory pressure at 5 cm H_2_O (1 cm H_2_O = 0.098 kp-A). The inhalation-to-breath ratio was 1:2. The inhaled oxygen concentration fraction was 0.6. After intubation, the patient was placed in the left lateral position. At the beginning of the procedure, we simultaneously established CO_2_ artificial pneumothorax and single-lung ventilation, maintaining the intrathoracic pressure at 8–10 mmHg. During the thoracoscopic phase, PetCO_2_ was maintained within the target range by adjusting the tidal volume and respiratory rate. Artificial pneumothorax was discontinued, and double-lung ventilation was resumed following intrathoracic esophageal free dissection and lymph node dissection.

Anesthesia maintenance: propofol 3 mg/(kg·h) and remifentanil 10–20 μg/(kg·h) were administered via continuous intravenous infusion, rocuronium 0.2 mg/kg was administered intermittently via intravenous injection, and 1–2% sevoflurane was administered via inhalation to maintain the BIS value between 40 and 60. During surgery, each patient was insulated with an inflatable thermal blanket to maintain body temperature at approximately 37 °C. After stabilization of vital signs and respiratory recovery, the tracheal catheter was removed, and the patients were transferred to the post-anesthesia care unit for follow-up diagnosis and treatment. All patients who underwent surgery were administered intravenous controlled analgesia: sufentanil (100 μg+), ondansetron (16 mg), and butorphanol (5 mg), diluted to 100 ml in a medical-grade 0.9% sodium chloride injection. Parameter settings included no background infusion, a basal rate of 3 ml/h, a patient-controlled analgesia dose of 0.5 ml, and a lockout interval of 15 min.

### Data collection

The clinical characteristics and demographic data of all patients were recorded, including age, sex, BMI, MMSE score, surgery time, ASA classification, preoperative hemoglobin concentration, total fluid infusion volume during surgery, intraoperative blood transfusion volume, intraoperative bleeding volume, intraoperative urine volume, and digital rating scale score for the first 3 days after surgery.

### Primary endpoint

Patients completed a preoperative cognitive status assessment based on the MMSE score prior to surgery. The main outcome was the incidence of delirium within the first 3 days after surgery.

The researchers underwent professional training before participating in the study, and the patients were evaluated by medical personnel who were proficient in the Confusion Assessment Method for the Intensive Care Unit (CAM-ICU). Two assessments were conducted twice (8:00–10:00 am and 18:00–20:00 pm) for 3 days post-surgery. The CAM-ICU [16] criteria comprise four aspects: acute changes or repeated fluctuations in consciousness status, attention deficit, thinking disorder, and changes in consciousness clarity. A positive delirium diagnosis was determined by the presence of the first two features, combined with either the third or fourth feature.

### Secondary endpoints

#### Enzyme-linked immunosorbent assay

Before intervention (T0) and at 1–3 days post-surgery (T1–T3), peripheral venous blood samples were collected from the patients and centrifuged at 3000 × g for 15 min to separate the serum. The serum was stored at −80 °C until analysis. The concentration of IL-1β was determined using an enzyme-linked immunosorbent assay kit (Thermo Fisher, USA), in accordance with the manufacturer’s instructions.

#### Quantitative reverse transcription-polymerase chain reaction (qRT-PCR) analysis

**Fig. 1:**
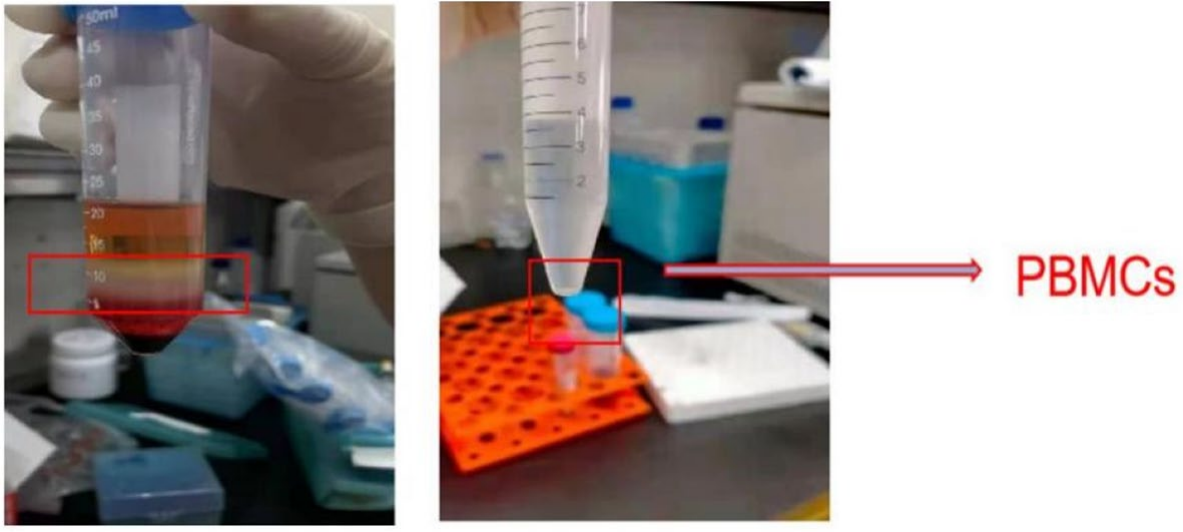
Extraction of peripheral blood mononuclear cells

A total of 3 ml of venous blood was collected from patients at T0–T3 into EDTA anticoagulant tubes. Human peripheral blood was mixed with lymphocyte separation solution (Sigma, USA) at a 1:1 ratio and centrifuged (LB-5000 medical centrifuge; Shanghai Zhaodi Biotechnology Co., Ltd.) at 3000 g/m for 10 min to separate peripheral blood mononuclear cells (Fig. 1). TRIzol reagent (Invitrogen) was added to lyse the cells and total RNA was extracted. RNasin was added to samples with OD260/280 values between 1.6 and 2.0, and the samples were stored in a −80 °C freezer until analysis. Next, 1 μg RNA was used as a template for cDNA synthesis. The reaction mixture volume was 20 μl. The reaction conditions were as follows: 37 °C for 60 min and 95 °C for 5 min. The obtained cDNA was stored at −20 °C. The experiment was performed according to the instructions of a reverse-transcription kit (Shanghai Sangong Biotechnology Co., LTD). The primers were designed and synthesized by Shanghai Sangong Biotechnology Co., LTD. The primer sequences are shown in Table 1. Real-time PCR was performed using a PCR kit (Shanghai Sangong Biotechnology Co., LTD) with a reaction system of volume 20 μl containing 10 μl of 2 × real-time PCR buffer, 0.4 μl of upstream and downstream primers, 2 μl of cDNA template, and 20 μl of water treated with diethylpyrocarbonate. The reaction conditions were as follows: pre-denaturation at 95 °C for 5 min, denaturation at 93 °C for 20 s, annealing at 56 °C for 20 s, and extension at 75 °C for 30 s (40 cycles). Two replicates were performed for each sample, with GAPDH as the internal reference gene. The results were analyzed using the CFX Manager Dx Software version 3.0. The expression of the target gene was reflected by the integrated optical density value, and the fold of expression change was calculated using 2^−ΔΔCt^ (ΔΔCt = average ΔCt of Group I − average ΔCt of Group C). The 2^−ΔΔCt^ method was used to determine the expression level of the target gene relative to that of the reference gene [17]. The primer sequences used for gene amplification are listed in Table 1.

**Table 1:** Sequence of primers used for gene amplification

Gene	Primer sequence (5′ → 3′)
*NLRP3* (NCBI: MK697677.1)	F: CTCTGCTCAGCACCACGAG
	R: CTCCACATGCCGAGGATGG
*Caspase-1* (NCBI: NM033294.3)	F: CCTGCCGTGGTGATAATGTT
	R: TCCACATCACAGGAACAGGC
*GAPDH* (NCBI: NM_002046.7)	F: GGAGCGAGATCCCTCCAAAAT
	R: GGCTGTTGTCATACTTCTCATGG

All primers were designed based on published sequences and synthesized by Sangon Biotech Co., Ltd. (Shanghai, China)

#### Adverse reactions

A continuous blood glucose monitoring system (Changsha Sinocare Biosensing Co., Ltd., China) was used to dynamically monitor blood glucose levels during the study period. Based on the American Diabetes Association dynamic blood glucose standard, a blood glucose level below 3.9 mmol/l was defined as hypoglycemia [18]. The blood glucose meter was used to record the time of hypoglycemia and the average blood glucose level.

### Statistical analysis

At our hospital, over the past 3 years, the incidence of POD among older patients undergoing radical resection for esophageal cancer has reached 48%. In the pre-experiment, the incidence of POD was 10% after intranasal administration of 40 U of long-acting insulin. A significance level of *α* = 0.05 (two-tailed) and a power of 1 − *β* = 0.9 were used to calculate the required sample. Power Analysis and Sample Size (PASS) software, version 11.0 (NCSS Statistical Software, Kaysville, UT, USA) was used, yielding a sample size of 50. To account for an anticipated 20% loss to follow-up, the sample size was adjusted to 60 patients.

Data analysis was performed using IBM SPSS Statistics version 26.0 (IBM Corp., Armonk, NY, USA). Analyses were based on the intent-to-treat outcomes. For quantitative data, the Shapiro–Wilk test and Levene’s test were used to examine the normal distribution and homogeneity of variance. Quantitative data that conform to normal distribution were expressed as mean ± standard deviation and were analyzed using an independent sample *t*-test. Quantitative data that do not follow a normal distribution were presented as median (interquartile range) and were analyzed using the Mann–Whitney *U* test. Count data were presented as frequency and percentage and were analyzed using the χ^2^ test or Fisher’s exact test. Repeated measures analysis of variance was used to determine the significance of differences in the average NLRP3 mRNA and caspase-1 mRNA expression and IL-1β concentration over time among the groups. Comparisons between the groups were performed using Dunnett’s *t*-test. The correlation between the changes in inflammatory markers (average NLRP3 mRNA, caspase-1 mRNA expression, and IL-1β concentration relative to baseline at different time points) and the incidence of postoperative delirium was tested using the point-biserial correlation coefficient. For all tests, results with *P* < 0.05 were considered statistically significant.

## Results

### Participants’ characteristics

**Fig. 2:**
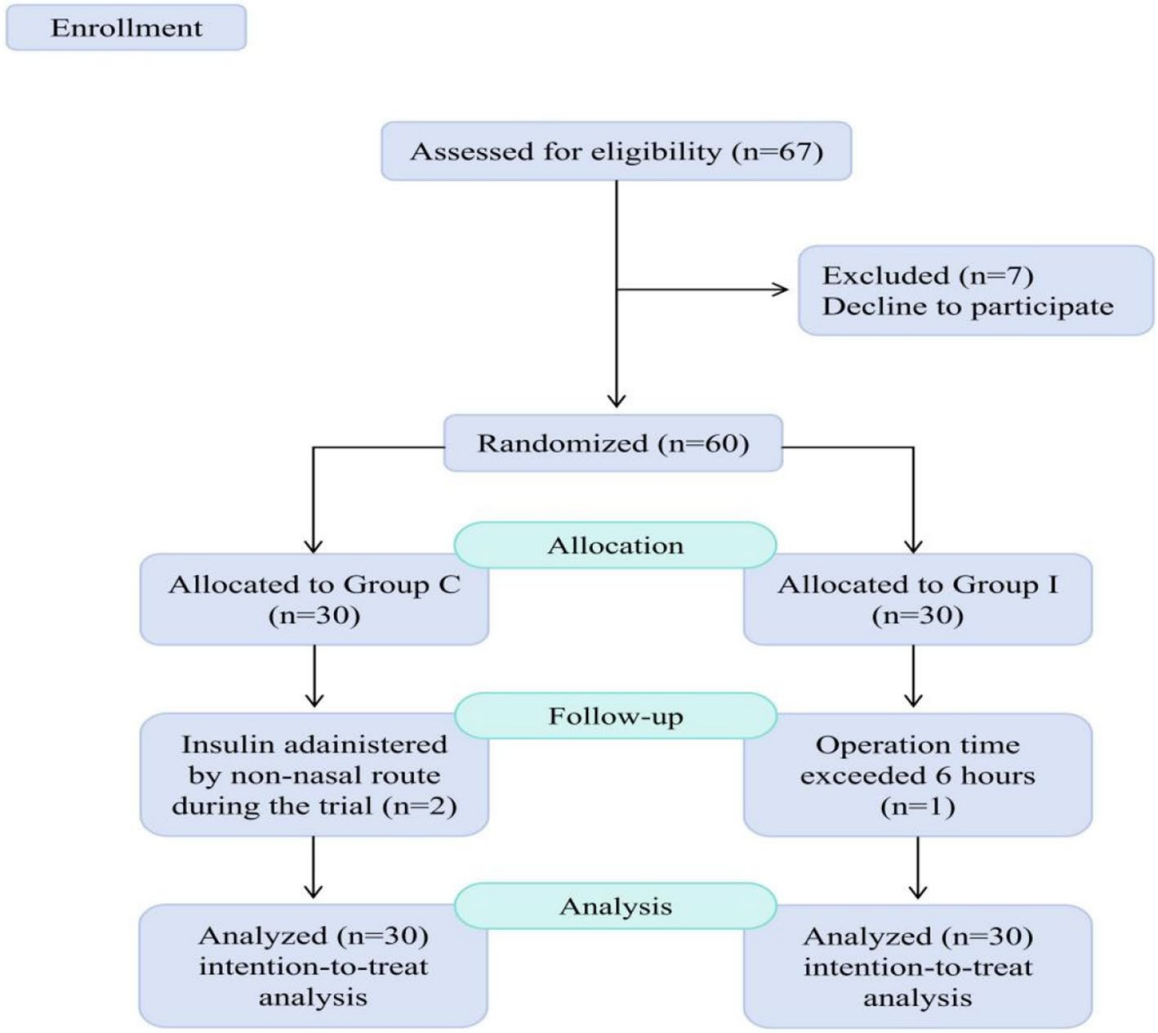
Study flow diagram

A total of 67 patients were screened for eligibility. Of these, seven declined to participate and were subsequently excluded. The remaining 60 eligible patients were randomly assigned to the control group (Group C, *n* = 30) or the insulin group (Group I, *n* = 30). Three patients were excluded from the per-protocol analysis: two in Group C who received insulin through alternative routes and one in Group I who underwent surgery with a duration of over 6 h. However, all 60 randomized patients were included in the intention-to-treat analysis (Fig. 2). Baseline demographic and clinical characteristics were comparable between groups, confirming successful randomization (Table 2).

**Table 2:** Patient demographics and clinical data

Characteristic	Group C (*n* = 30)	Group I (*n* = 30)	*P *value
Age (years)	67 (6)	68.5 (7)	0.306^$^
Sex (male)	27 (90%)	25 (83.3%)	0.706^*^
Body mass index (kg/m^2^)	21.90 ± 2.86	23.06 ± 2.59	0.105^&^
Education level			0.485^*^
Illiterate	25 (83.3%)	26 (86.7%)	
Primary	2 (6.7%)	3 (10%)	
Secondary	3 (10%)	1 (3.3%)	
MMSE score	21.00 (3)	21.00 (2)	0.706^$^
ASA classification			0.605^#^
II	17 (56.7%)	15 (50%)	
III	13 (43.3%)	15 (50%)	
Operation time (min)	265.93 ± 54.35	265.83 ± 37.63	0.993^&^
Preoperative hemoglobin concentration (g/l)	124.90 ± 11.75	129.00 ± 11.71	0.181^&^
Total intraoperative infusion (ml)	2600 (600)	2300 (925)	0.733^$^
Intraoperative blood loss (ml)	200 (200)	200 (100)	0.318^$^
Urine output (ml)	200 (113)	200 (200)	0.400^$^
NRS score			
T1	2.50 (1)	3.00 (1)	0.447^$^
T2	1.50 (1)	2.00 (1)	0.650^$^
T3	1.00 (1)	1.00 (1)	0.861^$^

Data are presented as mean ± SD, median (interquartile range), or percentage (proportion)

*Abbreviations*: *T1* postoperative day 1, *T2* postoperative day 2, *T3* postoperative day 3, *MMSE* Mini-Mental State Examination, *ASA* American Society of Anesthesiologists, *NRS* numerical rating scale

^$^Mann–Whitney U test

^*^Fisher’s exact test

^&^independent *t*-test

^#^Pearson χ^2^ test

### Prevalence of POD

Patients who received preoperative intranasal insulin exhibited a lower incidence of POD across multiple time points compared with those in the control group. On postoperative day 1, the incidence of POD was significantly reduced in Group I at both morning and evening assessments (16.7% vs. 46.7%, *P* = 0.012 and 16.7% vs. 43.3%, *P* = 0.024, respectively). This protective effect persisted on day 2, with Group I again showing fewer cases in both time periods (13.3% vs. 36.7%, *P* = 0.037 and 6.7% vs. 26.7%, *P* = 0.038). Although differences on day 3 were not statistically significant, the cumulative incidence of POD over the first three postoperative days remains significantly lower in the insulin group (16.7% vs. 46.7%, *P* = 0.012) (Table 3). These results suggest that intranasal insulin pretreatment may offer short-term neuroprotective benefits in preventing POD among elderly patients.

**Table 3:** Incidence of POD during the first three postoperative days

Postoperative day	Group C (*n* = 30)	Group I (*n* = 30)	cOR (95% CI)	*P *value
1 AM	14 (46.7%)	5 (16.7%)	4.38 (1.32–14.50)	0.012^a#^
1 PM	13 (43.3%)	5 (16.7%)	3.82 (1.15–12.71)	0.024^a#^
2 AM	11 (36.7%)	4 (13.3%)	3.76 (1.04–13.65)	0.037^a#^
2 PM	8 (26.7%)	2 (6.7%)	5.09 (0.98–26.43)	0.038^a#^
3 AM	4 (13.3%)	1 (3.3%)	4.46 (0.47–42.51)	0.351*
3 PM	2 (6.7%)	1 (3.3%)	2.07 (0.18–24.15)	1.00*
Within 3 days of surgery	14 (46.7%)	5 (16.7%)	4.38 (1.32–14.50)	0.012^a#^

Data are presented as percentage (proportion)

*Abbreviations*: *POD* postoperative delirium

^a^*P* < 0.05 for comparison between two groups

^#^Pearson χ^2^ test

*Fisher’s exact test

### Comparison of NLRP3 mRNA and caspase-1 mRNA expression levels and IL-1β concentration between the groups at different time points

Compared with Group C, the expression of NLRP3 mRNA and caspase-1 mRNA and the concentration of IL-1β were significantly downregulated in Group I at T1, T2, and T3 (*P* < 0.05). Compared to T0, the expression of NLRP3 mRNA and caspase-1 mRNA and the concentration of IL-1β were upregulated in both patient groups at T1, T2, and T3, with significant differences observed (*P* < 0.05) (Table 4). These findings imply that intranasal insulin may suppress peripheral inflammatory responses, possibly through inhibition of the NLRP3/caspase-1/IL-1β signaling pathway, which has been implicated in the pathogenesis of neuroinflammation and POD.

**Table 4:** Expression levels of key molecules in the NLRP3/caspase-1/IL-1β pathway

	Time point	Group C (*n* = 30)	Group I (*n* = 30)	*F* (group)/*P *value	*F* (time)*/P *value	*F* (group × time)*/P *value
NLRP3 mRNA	T0	25.40 ± 1.50	26.02 ± 2.26	*F* = 29.76 *P* < 0.001	*F* = 231.91 *P* < 0.001	*F* = 29.27 *P* < 0.001
	T1	35.66 ± 2.02^a^	30.88 ± 1.44^ab^			
	T2	31.41 ± 1.46^a^	28.80 ± 3.18^ab^			
	T3	28.04 ± 2.88^a^	27.61 ± 2.90^a^			
Caspase-1 mRNA	T0	23.98 ± 3.60	23.10 ± 3.37	*F* = 10.31 *P* = 0.004	*F* = 107.50 *P* < 0.001	*F* = 4.09 *P* = 0.002
	T1	31.05 ± 2.09^a^	28.70 ± 1.80^ab^			
	T2	28.09 ± 4.49^a^	25.67 ± 4.11^ab^			
	T3	25.12 ± 1.47	24.84 ± 1.92^a^			
IL-1β (pg/ml)	T0	31.01 ± 1.91	31.21 ± 2.10	*F* = 105.17 *P* < 0.001	*F* = 338.75 *P* < 0.001	*F* = 30.46 *P* < 0.001
	T1	51.39 ± 3.82^a^	43.15 ± 3.66^ab^			
	T2	45.32 ± 3.70^a^	37.73 ± 2.68^ab^			
	T3	34.80 ± 4.12^a^	34.05 ± 3.66^a^			

Data are expressed as mean ± SD. Comparisons between the groups were performed using the Dunnett *t*-test. Repeated measurements were compared using repeated measure ANOVA

*Abbreviations* *T0* before the first intervention, *T1* postoperative day 1, *T2* postoperative day 2, *T3* postoperative day 3

^a^*P* < 0.05 vs. T0

^b^*P* < 0.05 vs. Group C

* multiplication symbol

### Correlations between inflammatory biomarkers and POD

Correlation analyses revealed that in the control group, POD occurrence was significantly associated with increased levels of NLRP3 mRNA (*R*_pb_ = 0.449, *P* = 0.013), caspase-1 mRNA (*R*_pb_ = 0.546, *P* = 0.002), and IL-1β (*R*_pb_ = 0.424, *P* = 0.020) on postoperative day 1 (Table 5). In contrast, no such significant correlations were observed in the insulin group at any time point. This suggests that the association between early postoperative inflammation and delirium was disrupted by insulin pretreatment.

**Table 5:** The correlation between changes in inflammatory markers and postoperative delirium

		Time point	*R*_pb_ value	*P *value
NLRP3 mRNA	Group C (*n* = 30)	ΔT1	0.449	0.013
		ΔT2	0.033	0.864
		ΔT3	−0.057	0.764
	Group I (*n* = 30)	ΔT1	−0.019	0.920
		ΔT2	0.19	0.314
		ΔT3	0.078	0.683
Caspase-1 mRNA	Group C (*n* = 30)	ΔT1	0.546	0.002
		ΔT2	−0.356	0.054
		ΔT3	0.000	0.999
	Group I (*n* = 30)	ΔT1	−0.2	0.289
		ΔT2	0.026	0.891
		ΔT3	−0.238	0.206
IL-1β (pg/ml)	Group C (*n* = 30)	ΔT1	0.424	0.02
		ΔT2	−0.057	0.766
		ΔT3	0.210	0.266
	Group I (*n* = 30)	ΔT1	−0.336	0.069
		ΔT2	0.284	0.128
		ΔT3	−0.056	0.769

*R*_pb_, point-biserial correlation coefficient

ΔT1: T1 − T0; ΔT2: T2 − T0; ΔT3: T3 − T0

*Abbreviations*: *T0* before the first intervention, *T1* postoperative day 1, *T2* postoperative day 2, *T3* postoperative day 3

### Adverse events

No episodes of hypoglycemia were reported in either group throughout the study period. Mean blood glucose levels remained stable and comparable between the two groups before the first intervention and during the entire observation period (*P* > 0.05) (Table 6). These data confirm that intranasal delivery of rapid-acting insulin is safe and well tolerated in elderly surgical patients, without exerting significant effects on systemic glucose homeostasis.

**Table 6:** Average blood glucose values at different time points (mmol/l)

Time point	Group C (*n* = 30)	Group I (*n* = 30)	*P *value
T0	5.41 + 0.28	5.35 + 0.30	0.406
T4	5.70 + 0.43	5.82 + 0.44	0.286

Data are expressed as mean ± SD. Comparisons between the groups were performed using the independent *t*-test

*Abbreviations*: *T0* before the first intervention, *T4* the period from the first intervention to the last intervention

## Discussion

Postoperative delirium (POD) poses a significant clinical challenge in elderly patients undergoing major surgery, such as esophagectomy, and is associated with substantial morbidity [19, 20]. In this study, the 46.7% incidence of POD observed in our control cohort reaffirms the high burden of this complication in this vulnerable population [10].

Building upon our previous work, this study extends the investigation both methodologically and mechanistically. While prior trials validated a multiple-dose regimen of rapid-acting insulin for POD prevention [10, 11], the current study evaluated a single preoperative dose of long-acting insulin detemir. In a newly enrolled patient cohort, this simplified regimen confirmed a protective effect against POD. Mechanistically, it provides the first clinical evidence that this pretreatment reduces NLRP3 and caspase-1 mRNA expression in peripheral blood mononuclear cells, along with a decrease in serum IL-1β. Exploratory analysis further revealed that the postoperative rise in these inflammatory markers was associated with POD in controls, an association absent in the insulin-treated group. These findings demonstrate that the intervention leads to two parallel outcomes: a reduction in POD incidence and a downregulation of the peripheral NLRP3/caspase-1/IL-1β pathway. The absence of the association between these outcomes in the insulin group suggests a plausible mechanistic interaction; however, the present study design cannot establish a direct causal link or temporal sequence. Nevertheless, our findings implicate modulation of the NLRP3/caspase-1/IL-1β pathway as a potential mechanism underlying the observed clinical benefit. While the present design precludes causal inference, this study offers novel, pathway-specific evidence linking single-dose long-acting insulin to inflammasome inhibition and reduced delirium risk.

Notably, although chronic regular insulin administration may confer greater cognitive benefit than detemir in other contexts [21], the single-dose, preoperative detemir regimen employed here proved effective for POD prophylaxis. Its protective trend appears comparable to that achieved with prior multi-dose, rapid-acting insulin regimens, underscoring the utility of long-acting formulations for perioperative use. The choice of agent and regimen should be guided by specific clinical objectives. Administering a single dose on the day before surgery was designed for clinical practicality, aligning with typical admission timelines and the Enhanced Recovery After Surgery principle of efficient preoperative intervention [22]. This represents a substantial simplification over previous multi-dose protocols, potentially improving real-world adoption. While longer pretreatment might offer additional benefit, the current regimen balances pharmacological rationale with the imperative of clinical feasibility.

The inflammatory hypothesis is central to understanding POD pathogenesis [23, 24]. This process critically involves the activation of microglia, the resident immune cells of the CNS, which secrete pro-inflammatory cytokines such as IL-1β in response to stressors [25, 26]. Aging can prime microglia, increasing their susceptibility to a pro-inflammatory state and thereby contributing to neuroinflammation and cognitive vulnerability [13, 27, 28]. An upstream orchestrator of this inflammatory cascade is the NLRP3 inflammasome. This intracellular sensor complex, upon activation, cleaves pro-caspase-1 to its active form, which in turn processes the cytokine precursors pro-IL-1β and pro-IL-18 into their mature, bioactive forms, thereby amplifying the inflammatory signal [29, 30]. Preclinical studies strongly support its role in postoperative cognitive dysfunction, demonstrating that inhibition of the NLRP3/caspase-1 axis can mitigate anesthesia-induced neuroinflammation and cognitive impairment in aged animal models [13, 29, 31]. Our findings align with and extend this mechanistic model. The observation that insulin pretreatment attenuated the perioperative upregulation of the NLRP3/caspase-1/IL-1β axis in patient PBMCs, and concurrently dissociated its activity from delirium onset, positions this pathway as a plausible and modifiable target within POD’s neuroinflammatory cascade. While our study design does not delineate the precise molecular action of insulin, its known anti-inflammatory properties provide plausible mechanisms. Insulin signaling may exert its suppressive effect on the NLRP3 inflammasome through multiple upstream mechanisms. First, it can inhibit the nuclear factor kappa-B (NF-κB) pathway via the PI3K/Akt cascade [32], which is a master transcriptional regulator required for the expression of NLRP3 and pro-IL-1β [33]. Second, insulin has been shown to promote an anti-inflammatory macrophage phenotype [34], potentially reducing the cellular source of pro-inflammatory triggers. Finally, by improving cellular metabolic homeostasis and mitigating metabolic stress (e.g., oxidative stress)—a well-known activator of the NLRP3 inflammasome [35]—insulin may prevent the initial signal that primes and activates the inflammasome complex [36].

Thus, the insulin-induced modulation we observed provides a tangible molecular link between its clinical benefit in reducing POD incidence and the established inflammatory theory of delirium. Although our measurements were confined to the periphery, the well-documented crosstalk between the systemic and central immune systems underpins their relevance. Perioperative stress can compromise the integrity of the blood-brain barrier, potentially facilitating the influence of peripheral inflammatory mediators on the CNS [37, 38]. In addition, communication also occurs via humoral routes and neural pathways, such as the vagus nerve [39–42]. Consequently, peripheral inflammation is frequently correlated with central dysfunction and cognitive decline [38, 43–48]. In this context, PBMCs function as active immune sentinels rather than passive bystanders. Their expression of inflammasome components and cytokines reflects the systemic immune status and may offer an accessible window into the broader inflammatory milieu that impacts the brain [49, 50]. Therefore, the insulin-induced modulation of the NLRP3 pathway in PBMCs, as demonstrated here, may signify a systemic immunomodulatory effect with potential downstream consequences for neuroinflammation. This not only strengthens the rationale for targeting this pathway in POD prevention but also highlights PBMC-derived markers as valuable tools for future mechanistic and clinical investigation.

### Study limitations

Three main limitations should be considered. First, our investigation of inflammatory mechanisms was restricted to the transcriptional level of the NLRP3/caspase-1/IL-1β pathway in peripheral blood mononuclear cells (PBMCs). The absence of protein-level data and direct assessments of central nervous system inflammation precludes a comprehensive understanding of inflammasome activation in vivo. Second, the single-center design and relatively small sample size may affect the generalizability of our findings, necessitating future validation in larger, multi-center cohorts. Finally, while the CAM-ICU is a standard tool for POD diagnosis, it does not differentiate between clinical subtypes (e.g., hyperactive, hypoactive, or mixed). Consequently, our analysis could not address potential phenotypic differences, which may be associated with distinct pathophysiological mechanisms and outcomes.

## Conclusion

In conclusion, a single preoperative dose of intranasal insulin effectively reduces the incidence of postoperative delirium and mitigates the peripheral inflammatory response in elderly patients undergoing radical esophagectomy. The observed clinical benefit is consistent with the downregulation of the NLRP3/caspase-1 signaling pathway. Notably, insulin pretreatment abolished the significant correlation seen in controls between postoperative activation of this pathway and delirium onset, suggesting that disruption of this specific inflammatory link may be a key mechanism contributing to its protective effect.

## Data Availability

The raw data for this study are available from the corresponding author on reasonable request.

## Abbreviations

BIS: Bispectral index
BMI: Body mass index
ChIV: Chitohexaose
CONSORT: Comprehensive Standards for Trial Reporting
CAM-ICU: Confusion Assessment Method for the Intensive Care Unit
EC: Esophageal cancer
IL: Interleukin
MMSE: Mini-Mental State Examination
NLRP3: Nucleoside-binding oligomerization domain-like receptor protein 3
POD: Postoperative delirium
PASS: Power Analysis and Sample Size
qRT-PCR: Quantitative reverse transcription-polymerase chain reaction
ERAS: Enhanced Recovery After Surgery

## References

[1] Satapathy P, Gaidhane AM, Vadia N, et al. Prevalence of recurrent nerve injury among esophageal cancer patients undergoing esophagectomy: a systematic review and meta-analysis. Surg Open Sci. 2025;27:68–80. 10.1016/j.sopen.2025.05.009. 40697900 10.1016/j.sopen.2025.05.009 PMC12282457

[2] Baranov NS, Slootmans C, van Workum F, Klarenbeek BR, Schoon Y, Rosman C. Outcomes of curative esophageal cancer surgery in elderly: a meta-analysis. World J Gastrointest Oncol. 2021;13(2):131–46. 10.4251/wjgo.v13.i2.131. 33643529 10.4251/wjgo.v13.i2.131 PMC7896422

[3] Thedim M, Vacas S. Anesthetic sensitivity and resilience in the aging brain: implications for perioperative neurocognitive disorders. Anesthesiol Perioper Sci. 2025;3:11. 10.1007/s44254-025-00094-6.

[4] Urbánek L, Urbánková P, Satinský I, Trávníček T, Penka I, Hruda J. Postoperative delirium. Rozhl Chir. 2023;102(10):381–6. 10.33699/PIS.2023.102.10.381-386. 38302424 10.33699/PIS.2023.102.10.381-386

[5] Foley KA, Djaiani G. Update of the European Society of Anaesthesiology and Intensive Care Medicine evidence-based and consensus-based guideline on postoperative delirium in adult patients. Eur J Anaesthesiol. 2025;42(1):86–7. 10.1097/EJA.0000000000002043. 39628423 10.1097/EJA.0000000000002043

[6] Fenaroli F, Valerio A, Ingrassia R. Ischemic neuroprotection by insulin with down-regulation of divalent metal transporter 1 (DMT1) expression and ferrous iron-dependent cell death. Biomolecules. 2024;14(7):856. 10.3390/biom14070856. 39062570 10.3390/biom14070856 PMC11274861

[7] Arda DB, Tunç KC, Bozkurt MF, Bora ES, Çiğel A, Erbaş O. Intranasal insulin eases autism in rats via GDF-15 and anti-inflammatory pathways. Curr Issues Mol Biol. 2024;46(9):10530–44. 10.3390/cimb46090624. 39329976 10.3390/cimb46090624 PMC11431515

[8] Shpakov AO, Zorina II, Derkach KV. Hot spots for the use of intranasal insulin: cerebral ischemia, brain injury, diabetes mellitus, endocrine disorders and postoperative delirium. Int J Mol Sci. 2023;24(4):3278. 10.3390/ijms24043278. 36834685 10.3390/ijms24043278 PMC9962062

[9] Badenes R, Qeva E, Giordano G, Romero-García N, Bilotta F. Intranasal insulin administration to prevent delayed neurocognitive recovery and postoperative neurocognitive disorder: a narrative review. Int J Environ Res Public Health. 2021;18(5):2681. 10.3390/ijerph18052681. 33799976 10.3390/ijerph18052681 PMC7967645

[10] Huang Q, Shi Q, Yi X, et al. Effect of repeated intranasal administration of different doses of insulin on postoperative delirium, serum τ and Aβ protein in elderly patients undergoing radical esophageal cancer surgery. Neuropsychiatr Dis Treat. 2023;19:1017–26. 10.2147/NDT.S405426. 37144143 10.2147/NDT.S405426 PMC10153451

[11] Huang Q, Li Q, Qin F, et al. Repeated preoperative intranasal administration of insulin decreases the incidence of postoperative delirium in elderly patients undergoing laparoscopic radical gastrointestinal surgery: a randomized, placebo-controlled, double-blinded clinical study. Am J Geriatr Psychiatry. 2021;29(12):1202–11. 10.1016/j.jagp.2021.02.043. 33757723 10.1016/j.jagp.2021.02.043

[12] Li J, Li L, He J, Xu J, Bao F. The NLRP3 inflammasome is a potential mechanism and therapeutic target for perioperative neurocognitive disorders. Front Aging Neurosci. 2023;14:1072003. 10.3389/fnagi.2022.1072003. 36688154 10.3389/fnagi.2022.1072003 PMC9845955

[13] Wang Z, Meng S, Cao L, Chen Y, Zuo Z, Peng S. Critical role of NLRP3-caspase-1 pathway in age-dependent isoflurane-induced microglial inflammatory response and cognitive impairment. J Neuroinflammation. 2018;15(1):109. 10.1186/s12974-018-1137-1. 29665808 10.1186/s12974-018-1137-1 PMC5904978

[14] Zhao S, Chen F, Wang D, Han W, Zhang Y, Yin Q. NLRP3 inflammasomes are involved in the progression of postoperative cognitive dysfunction: from mechanism to treatment. Neurosurg Rev. 2021;44(4):1815–31. 10.1007/s10143-020-01387-z. 32918635 10.1007/s10143-020-01387-z

[15] Claxton A, Baker LD, Hanson A, et al. Long-acting intranasal insulin detemir improves cognition for adults with mild cognitive impairment or early-stage Alzheimer’s disease dementia. J Alzheimers Dis. 2015;44(3):897–906. 10.3233/JAD-141791. 25374101 10.3233/JAD-141791

[16] Rieck KM, Pagali S, Miller DM. Delirium in hospitalized older adults. Hosp Pract (1995). 2020;48(sup1):3–16. 10.1080/21548331.2019.1709359. 31874064 10.1080/21548331.2019.1709359

[17] Livak KJ, Schmittgen TD. Analysis of relative gene expression data using real-time quantitative PCR and the 2(-delta delta C(T)) method. Methods. 2001;25(4):402–8. 10.1006/meth.2001.1262. 11846609 10.1006/meth.2001.1262

[18] Frias JP, Deenadayalan S, Erichsen L, et al. Efficacy and safety of co-administered once-weekly cagrilintide 2·4 mg with once-weekly semaglutide 2·4 mg in type 2 diabetes: a multicentre, randomised, double-blind, active-controlled, phase 2 trial. Lancet. 2023;402(10403):720–30. 10.1016/S0140-6736(23)01163-7. 37364590 10.1016/S0140-6736(23)01163-7

[19] Qureshi O, Arthur ME. Recent advances in predicting, preventing, and managing postoperative delirium. Fac Rev. 2023;12:19. 10.12703/r/12-19. 37529149 10.12703/r/12-19 PMC10388843

[20] Sato H, Miyawaki Y, Lee S. [ESSENSE concept and perioperative management in esophageal cancer treatment]. Kyobu Geka. 2023;76(10):904–7. 38056860

[21] Craft S, Claxton A, Baker LD, et al. Effects of regular and long-acting insulin on cognition and Alzheimer’s disease biomarkers: a pilot clinical trial. J Alzheimers Dis. 2017;57(4):1325–34. 10.3233/JAD-161256. 28372335 10.3233/JAD-161256 PMC5409050

[22] Powers BK, Ponder HL, Findley R, Wolfe R, Patel GP, Parrish RH 2nd. Enhanced recovery after surgery (ERAS®) society abdominal and thoracic surgery recommendations: a systematic review and comparison of guidelines for perioperative and pharmacotherapy core items. World J Surg. 2024;48(3):509–23. 10.1002/wjs.12101. 38348514 10.1002/wjs.12101

[23] Tang Y, Le W. Differential roles of M1 and M2 microglia in neurodegenerative diseases. Mol Neurobiol. 2016;53(2):1181–94. 10.1007/s12035-014-9070-5. 25598354 10.1007/s12035-014-9070-5

[24] Thedim M, Vacas S. Anesthetic sensitivity and resilience in the aging brain: implications for perioperative neurocognitive disorders. Anesthesiology and Perioperative Science. 2025;3:11.

[25] Norden DM, Godbout JP. Review: microglia of the aged brain: primed to be activated and resistant to regulation. Neuropathol Appl Neurobiol. 2013;39(1):19–34. 10.1111/j.1365-2990.2012.01306.x. 23039106 10.1111/j.1365-2990.2012.01306.x PMC3553257

[26] Franceschi C, Campisi J. Chronic inflammation (inflammaging) and its potential contribution to age-associated diseases. J Gerontol A Biol Sci Med Sci. 2014;69(1):S4-9. 10.1093/gerona/glu057. 24833586 10.1093/gerona/glu057

[27] van den Boogaard M, Slooter AJ, Brüggemann RJ, et al. Prevention of ICU delirium and delirium-related outcome with haloperidol: a study protocol for a multicenter randomized controlled trial. Trials. 2013;14:400. 10.1186/1745-6215-14-400. 24261644 10.1186/1745-6215-14-400 PMC4222562

[28] Walker KA, Basisty N, Wilson DM 3rd, Ferrucci L. Connecting aging biology and inflammation in the omics era. J Clin Invest. 2022;132(14):e158448. 10.1172/JCI158448. 35838044 10.1172/JCI158448 PMC9282936

[29] Shao A, Fei J, Feng S, Weng J. Chikusetsu saponin IVa alleviated sevoflurane-induced neuroinflammation and cognitive impairment by blocking NLRP3/caspase-1 pathway. Pharmacol Rep. 2020;72(4):833–45. 10.1007/s43440-020-00078-2. 32124392 10.1007/s43440-020-00078-2

[30] Cho I, Koo BN, Kim SY, et al. Neuroprotective effect of dexmedetomidine against postoperative cognitive decline via NLRP3 inflammasome signaling pathway. Int J Mol Sci. 2022;23(15):8806. 10.3390/ijms23158806. 35955939 10.3390/ijms23158806 PMC9369249

[31] Schneider E, Spetter MS, Martin E, Thomas JM, Lee M, Hallschmid M, et al. The effect of intranasal insulin on appetite and mood in women with and without obesity: an experimental medicine study. Int J Obes (Lond). 2022;46(7):1319–27. 10.1038/s41366-022-01115-1. 35397638 10.1038/s41366-022-01115-1 PMC9239904

[32] Dandona P, Aljada A, Bandyopadhyay A. Inflammation: the link between insulin resistance, obesity and diabetes. Trends Immunol. 2004;25(1):4–7. 10.1016/j.it.2003.10.013. 14698276 10.1016/j.it.2003.10.013

[33] Bai B, Yang Y, Wang Q, Li M, Tian C, Liu Y, et al. NLRP3 inflammasome in endothelial dysfunction. Cell Death Dis. 2020;18(9):776. 10.1038/s41419-020-02985-x. 10.1038/s41419-020-02985-x PMC7501262 32948742

[34] Yu T, Gao M, Yang P, Liu D, Wang D, Song F, et al. Insulin promotes macrophage phenotype transition through PI3K/Akt and PPAR-γ signaling during diabetic wound healing. J Cell Physiol. 2019;234(4):4217–31. 10.1002/jcp.27185. 30132863 10.1002/jcp.27185

[35] Shimada K, Crother TR, Karlin J, Dagvadorj J, Chiba N, Chen S, et al. Oxidized mitochondrial DNA activates the NLRP3 inflammasome during apoptosis. Immunity. 2012. 10.1016/j.immuni.2012.01.009. 22342844 10.1016/j.immuni.2012.01.009 PMC3312986

[36] Chang YW, Hung LC, Chen YC, Wang WH, Lin CY, Tzeng HH, et al. Insulin reduces inflammation by regulating the activation of the NLRP3 inflammasome. Front Immunol. 2021;11:587229. 10.3389/fimmu.2020.587229. 33679687 10.3389/fimmu.2020.587229 PMC7933514

[37] Wang Y, Wang D, Yin K, et al. Lycopene attenuates oxidative stress, inflammation, and apoptosis by modulating Nrf2/NF-κB balance in sulfamethoxazole-induced neurotoxicity in grass carp (*Ctenopharyngodon idella*). Fish Shellfish Immunol. 2022;121:322–31. 10.1016/j.fsi.2022.01.012. 35032680 10.1016/j.fsi.2022.01.012

[38] Alam A, Hana Z, Jin Z, Suen KC, Ma D. Surgery, neuroinflammation and cognitive impairment. EBioMedicine. 2018;37:547–56. 10.1016/j.ebiom.2018.10.021. 30348620 10.1016/j.ebiom.2018.10.021 PMC6284418

[39] Lerman I, Davis B, Huang M, et al. Noninvasive vagus nerve stimulation alters neural response and physiological autonomic tone to noxious thermal challenge. PLoS ONE. 2019;14(2):e0201212. 10.1371/journal.pone.0201212. 30759089 10.1371/journal.pone.0201212 PMC6373934

[40] Yagi M, Morishita K, Ueno A, et al. Electrical stimulation of the vagus nerve improves intestinal blood flow after trauma and hemorrhagic shock. Surgery. 2020;167(3):638–45. 10.1016/j.surg.2019.09.024. 31759624 10.1016/j.surg.2019.09.024

[41] Chao CC, Yang WK, Yeh TY, et al. Transthyretin variants impact blood-nerve barrier and neuroinflammation in amyloidotic neuropathy. Brain. 2025;148(7):2537–50. 10.1093/brain/awaf028. 39874259 10.1093/brain/awaf028

[42] Förstermann U, Sessa WC. Nitric oxide synthases: regulation and function. Eur Heart J. 2012;33(7):829–37. 10.1093/eurheartj/ehr304. 21890489 10.1093/eurheartj/ehr304 PMC3345541

[43] D’Mello C, Swain MG. Immune-to-brain communication pathways in inflammation-associated sickness and depression. Curr Top Behav Neurosci. 2017;31:73–94. 10.1007/7854_2016_37. 27677781 10.1007/7854_2016_37

[44] Peng W, Lu W, Jiang X, et al. Current progress on neuroinflammation-mediated postoperative cognitive dysfunction: an update. Curr Mol Med. 2023;23(10):1077–86. 10.2174/1566524023666221118140523. 36411553 10.2174/1566524023666221118140523

[45] Yang XD, Wang LK, Wu HY, Jiao L. Effects of prebiotic galacto-oligosaccharide on postoperative cognitive dysfunction and neuroinflammation through targeting of the gut-brain axis. BMC Anesthesiol. 2018;18(1):177. 10.1186/s12871-018-0642-1. 30497394 10.1186/s12871-018-0642-1 PMC6267821

[46] Dahm T, Rudolph H, Schwerk C, Schroten H, Tenenbaum T. Neuroinvasion and inflammation in viral central nervous system infections. Mediators Inflamm. 2016;2016:8562805. 10.1155/2016/8562805. 27313404 10.1155/2016/8562805 PMC4897715

[47] Hoffmann O, Zipp F, Weber JR. Tumour necrosis factor-related apoptosis-inducing ligand (TRAIL) in central nervous system inflammation. J Mol Med (Berl). 2009;87(8):753–63. 10.1007/s00109-009-0484-x. 19449143 10.1007/s00109-009-0484-x

[48] Rodriguez-Smith J, Lin YC, Tsai WL, et al. Cerebrospinal fluid cytokines correlate with aseptic meningitis and blood-brain barrier function in neonatal-onset multisystem inflammatory disease: central nervous system biomarkers in neonatal-onset multisystem inflammatory disease correlate with central nervous system inflammation. Arthritis Rheumatol. 2017;69(6):1325–36. 10.1002/art.40055. 28118536 10.1002/art.40055 PMC5449229

[49] Fan Z, Pan YT, Zhang ZY, et al. Systemic activation of NLRP3 inflammasome and plasma α-synuclein levels are correlated with motor severity and progression in Parkinson’s disease. J Neuroinflammation. 2020;17(1):11. 10.1186/s12974-019-1670-6. 31915018 10.1186/s12974-019-1670-6 PMC6950934

[50] Szabo A, O’Connell KS, Akkouh IA, et al. Elevated levels of peripheral and central nervous system immune markers reflect innate immune dysregulation in autism spectrum disorder. Psychiatry Res. 2024;342:116245. 10.1016/j.psychres.2024.116245. 39481220 10.1016/j.psychres.2024.116245

